# Children’s palliative care now! Highlights from the second ICPCN conference on children’s palliative care, 18–21 May 2016, Buenos Aires, Argentina

**DOI:** 10.3332/ecancer.2016.667

**Published:** 2016-08-23

**Authors:** J Downing, R Kiman, S Boucher, B Nkosi, B Steel, C Marston, E Lascar, J Marston

**Affiliations:** 1 International Children’s Palliative Care Network, New Bond House, Bond Street, Bristol, UK; 2 Palliative Care, Makerere University, PO Box 7072, Kampala, Uganda.; 3 Paediatric Palliative Care, Hospital Nacional Prof A Posadas, Av Pres Arturo U Illia, Villa Sarmiento, Buenos Aires, Argentina; 4 International Children’s Palliative Care Network, Cluster Box 3050, Assagay, 3624, South Africa; 5 Hospital de Niños Dr Ricardo Gutiérrez, C1425EFD Autonomous City of Buenos Aires, Argentina

**Keywords:** palliative care, children, international, commitment, integration, education, research, Argentina, WHA resolution

## Abstract

The International Children’s Palliative Care Network held its second international conference on children’s palliative care in Buenos Aires, Argentina, from the 18th–21st May 2016. The theme of the conference was ‘*Children’s Palliative Care…. Now!*’ emphasising the need for palliative care for children now, as the future will be too late for many of them. Six pre-conference workshops were held, addressing issues connected to pain assessment and management, adolescent palliative care, ethics and decision-making, developing programmes, the basics of children’s palliative care, and hidden aspects of children’s palliative care. The conference brought together 410 participants from 40 countries. Plenary, concurrent, and poster presentations covered issues around the status of children’s palliative care, genetics, perinatal and neonatal palliative care, the impact of children’s palliative care and the experiences of parents and volunteers, palliative care as a human right, education in children’s palliative care, managing complex pain in children, spiritual care and when to initiate palliative care. The ‘Big Debate’ explored issues around decision-making and end of life care in children, and gave participants the opportunity to explore a sensitive and thought provoking topic. At the end of the conference, delegates were urged to sign the Commitment of Buenos Aires which called for governments to implement the WHA resolution and ensure access to palliative care for neonates, children and their families, and also commits us as palliative care providers to share all that we can and collaborate with each other to achieve the global vision of palliative care for all children who need it. The conference highlighted the ongoing issues in children’s palliative care and participants were continually challenged to ensure that children can access palliative care NOW.

## Introduction

The International Children’s Palliative Care Network (ICPCN) held its second international conference on children’s palliative care in Buenos Aires, Argentina. The conference was held at the Paediatric Teaching and Training Centre ‘Dr Carlos Gianantonio’ of the Argentinean Society of Paediatrics from the 18th–21st May 2016, with pre-conference workshops on the 18th May 2016.

Much emphasis has been placed on the development of palliative care, for both adults and children since the signing of the World Health Assembly Resolution on Palliative Care in May 2014 [1]. This is the first ever resolution on palliative care and recognises that palliative care is ‘*fundamental to improving quality of life, well-being, comfort and human dignity for individuals, being an effective person-centred health service that values patients’ need to received adequate, personally and culturally sensitive information on their health status, and their central role in making decisions about the treatment received*’ [1] (p2). The resolution urges member states to develop, strengthen, and implement palliative care for all in need, including children, through a wide range of activities including education and training, partnerships and sharing of experiences. Thus, ICPCN took the opportunity of holding its second conference in Latin America, to help promote and advocate for children’s palliative care within the region.

The ICPCN aims to promote the development of children’s palliative care around the world. It is the only global organisation working as a global action network in the field of children’s palliative care and is acknowledged as the lead for children’s palliative care by the World Health Organisation and other international agencies [2–3]. The success of the ICPCN is built upon the sum of its parts, as it is essential that members work together and collaborate in order to improve children’s access to palliative care throughout the world. At present, the network has over 350 organisational members and 1588 individual members from more than 100 countries. ICPCN’s vision is to live in a world where children’s palliative care is acknowledged and respected as a unique service and every child and young person with life-limiting or life-threatening conditions and their families can receive the best quality of life and care regardless of which country they live in. Their holistic needs encompass physical, emotional, spiritual, and developmental aspects of care and ICPCN’s mission is therefore to attain the best achievable quality of life and care for children and young people with life-limiting conditions, their families and carers worldwide by raising awareness of children’s palliative care, advocating for the global development of children’s palliative care services and sharing expertise, skills, and knowledge.

The provision of an international conference enables participants from around the world to learn from each other. Thus, it was anticipated that this second International conference, held in Argentina, would promote ICPCN’s mission, and also demonstrates the ongoing developments and evidence base for children’s palliative care, advocating for its development in Latin America and throughout the world.

## Palliative and cancer care for children in Argentina

Argentina is the second largest country in Latin America after Brazil. It had a population of 41 million people in 2012 with over 12 million children under the age of 18. There are around 4725 children living with HIV/AIDS, and over the past decade, the number of children infected has stabilised at 100 per year, with a rate of vertical transmission between 5% and 6% [4]. About 18,069 cancer cases were reported in children under 15 years of age between 2000 and 2013 [5] with leaukaemia being the most frequent malignancy in children, followed by central nervous system (CNS) tumours and lymphomas. This distribution is similar to that described in countries in Europe and North America. The population of children surviving cancer in low- and middle-income countries is less than 40% [6] and Latin America has around 12% of childhood cancers worldwide. In Argentina, the Argentinian Oncopaediatric Registry (ROHA) was launched in 2000 and absorbed by the Argentinian National Institute of Cancer in 2011. Data show that 75% of children in Argentina with cancer are treated at public institutions [7].

While it is acknowledged that palliative care should be provided for children with a wide range of life-limiting and life-threatening illnesses, cancer is one of the main illnesses requiring palliative care. Palliative care in Argentina has developed from the first few initiatives in the mid-1980s to more than 80 palliative care services throughout the country, with at least 16 of these providing palliative care for children [8–9]. The first children’s palliative care programme was developed in Argentina in 1985 within the Prager-Bild Foundation, and they, along with other programmes, became the pioneers in providing hospital-based palliative care support for children in Argentina [8]. In the past 10 years, there have been great strides in the development of children’s palliative care in Argentina with the provision of guidelines, postgraduate degrees and the formation of a Palliative Care Task Force. Recently, palliative care has been recognised as a medical speciality with paediatricians now able to specialist in children’s palliative care. However, while children’s palliative care is developing within the country, there are still an unacceptable number of children who do not have access to palliative care, thus the goal for the future is to increase human resources for the provision of children’s palliative care [8]. Research by the ICPCN has identified 98,470 children in Argentina who would benefit from palliative care.

## Pre-conference workshops

Six pre-conference workshops were held on Wednesday 18th May (Table 1). Over 150 participants attended the pre-conference workshops including doctors, nurses, social workers, volunteers, bereavement counsellors and religious leaders. While the majority of participants at the pre-conference workshops were from Argentina, the surrounding Latin American countries, such as Brazil, Uruguay, Chile, Colombia, and Costa Rica, were also well represented along with international participants. Many children with life-limiting or life-threatening illness experience medium to severe somatic, visceral, chronic, neuropathic, and/or spiritual pain. Pain may be disease related or treatment related; however, regardless of the cause, data reveal a significant under the treatment of pain in children receiving palliative care in both high- and low-income countries. For this reason, a pre-conference workshop was held on pain in children, with simultaneous translation in English and Spanish. This workshop was very popular with participants from a wide variety of disciplines. The workshop explored multimodal analgesia as effective treatment strategies in the management of common pain types in children suffering from advanced illnesses. Application of the World Health Organisation (WHO) principles of pain management [10] results in good pain relief for the majority of children with advanced disease. State-of-the-art pain management in the 21st century requires that pharmacological management must be combined with rehabilitative, psychological, and integrative (‘non-pharmacological’) therapies to manage a child’s pain effectively. At the end of the workshop, participants were encouraged to think about how they could improve pain control for the children in their care, and what needs to be done to accomplish this.

Adolescence, a period in human growth and development that occurs after childhood and before adulthood, represents one of the critical transitions in the life span and is characterised by a tremendous pace in growth and change that is second only to that of infancy. The provision of palliative care for adolescents is often seen as a challenge, with adolescents not being recognised as children, but also not yet adults [12]. Thus, one of the pre-conference workshops focused on caring for adolescents. Participants were able to share their own experiences and challenges throughout the day, and it was fascinating to hear of the practices of individuals from different settings and countries.

The other four pre-conference workshops were presented in either English or Spanish with two in the morning and another two in the afternoon. The workshop, *Principles of Children’s Palliative Care*, was directed at those new to the field and aimed to share the basic principles of children’s palliative care. A workshop on ‘*The invisible aspects of children’s palliative care*’ explored some of the less obvious aspects of children’s palliative care. Participants attending the workshop felt that it gave them the opportunity to explore issues that are often neglected within the provision of care and found it to be most useful and informative. *Ethics and decision-making* are pivotal issues within children’s palliative care; therefore, a pre-conference workshop was held to explore decision-making in four different areas. Small group discussions were held in each of these areas followed by feedback and discussion with the whole group. Important issues were raised and experiences shared between participants, which enabled them to think widely around the topics and possible scenarios. The final pre-conference workshop was about ‘*Developing children’s palliative care programmes*’ with facilitators sharing experiences from developing programmes in China, Belarus, and India. Time was spent in group discussion exploring some of the issues experienced by the participants in developing their own programmes, and participants were able to support each other and help each other think through some of the challenges and options open to them.

## Conference summary

The theme of the conference was *Children’s Palliative Care …. Now!* and was the culmination of the ICPCN’s 10th anniversary project, themed ‘Now for children with life-limiting conditions’. Stressing the fact that for all those children around the world needing palliative care—they need it now, as for many of them, tomorrow will be too late which is why ICPCN has been raising awareness of children’s palliative care and advocating for its development and provision around the world. The scientific programme included a variety of plenary sessions (12 papers) and debate, 58 oral breakout presentations, including workshops, 134 poster presentations, and six ‘meet the expert’ sessions. Oral and poster abstracts were accepted from 32 countries with representation from those new to the field and those who have been working in the field for many years, thus demonstrating the breadth of participants attending the conference. Workshops were held in areas such as spirituality and the Religion of the World Charter for Children’s Palliative Care (http://www.maruzza.org/en/religions-of-the-world-charter-for-childrens-palliative-care/), Advocacy, and electronic communication. Meet the expert sessions also covered areas such as bereavement, challenging communication, access to medication, managing complex pain, fundraising, and advocacy.

The conference, held at the paediatric teaching and training centre ‘Dr Carlos Gianantonio’ of the Argentine Society of Paediatrics in Buenos Aires, brought together 410 participants representing 40 countries from around the world (Table 2). The conference brought together a range of clinicians, advocates, academics, clergy, researchers, social workers, policy makers, Ministry of Health officials, volunteers, play therapists and others working in the field of children’s palliative care.

Delegates were welcomed in true Buenos Aires style at the opening ceremony on the evening of Wednesday 18th May. They were given a warm welcome by the conference chairs and from local dignitaries including a representative from the Argentine Ministry of Health, the President of the Sociedad Argentina De Pediatria, the presidents of the Latin American Association for Palliative Care (ALCP), and the Asociacion Argentina de Medicine y Cuidados Paliativos. The importance of hosting the conference in Argentina and the Latin American region was noted, and dignitaries stressed the need for and their commitment to, children’s palliative care both nationally and within the region. This support for children’s palliative care was reiterated in the messages of support for the conference sent in a letter by Her Royal Highness the Duchess of Cambridge, a letter from the Vatican acknowledging the value of the ICPCN Now campaign, a video message from Cape Town from Archbishop Emeritus Desmond Tutu, and a video message from ICPCN’s Global Ambassador Micheline Etkin. All spoke of the valuable work done by those working in children’s palliative care, with the Archbishop noting that ‘*when children suffer, God cries; when we help these children. we wipe Gods tears*’.

The scientific content of the conference got underway through the keynote address given by Dr Lisbeth Quesada from Costa Rica who has been at the forefront of the development of palliative care in Costa Rica and the Latin American region. She gave a passionate address, reflecting on the past and highlighting some of the lessons learnt in the development of children’s palliative care in the region that can be used in future developments, in both Latin America and internationally. She ended her presentation by urging participants to stand up for children’s palliative care NOW and to be advocates for the ongoing development of such services.

The first morning of the conference helped to set the scene for children’s palliative care, both globally and within the region. The meaning of children’s palliative care in the 21st Century was discussed and the need to reach children with life-limiting conditions in a rapidly changing world, with limited and insufficient resources and many programmes struggling to survive. Disparities between low- and high-income countries were pointed out and participants challenged to work together to integrate palliative care for children into health systems, to share resources and network to reach every child in need of palliative care—regardless of where they live, their race, gender, or religion. A video message from Dr Marie Charlotte Bouesseau, Advisor from the World Health Organisation (WHO), reiterated the need for children’s palliative care, highlighting the WHA resolution and the commitment from the WHO to promote palliative care for children around the world. The situation in Latin America was presented, sharing some of the results of the mapping exercise undertaken in order to develop the Atlas of Palliative Care in Latin America [13]. The last presentation in the opening plenary focused on child health in Argentina and the role of children’s palliative care within this setting.

Concurrent sessions then focused on several issues, including (1) Family and volunteers’ perspectives such as: How schools can support siblings; experiences from Zimbabwe, the Netherlands and Australia; and support for black and minority ethnic children in the United Kingdom, (2) The use of opioids and interventions for pain management in children, and (3) a workshop on Spirituality and the Religion of the World Charter for Children’s Palliative Care. This latter workshop, supported by the Maruzza Foundation, was an opportunity to discuss the importance of spirituality in children’s palliative care and local religious leaders were invited to attend the workshop in order to advocate for their commitment to children’s palliative care. Prior to lunch, the plenary session focused on the impact on society of a child with a life-limiting condition. Drawing on experiences within the field of law, public health, and ethics, the social responsibility that we all share as part of mankind was discussed in the context of palliative care for children as a human right ‘Homo sum, humani nihil a me alienu, puto’ [‘I’m a man, and everything humane is my concern’. (Terencio)].

Concurrent sessions in the afternoon focused on perinatal and neonatal palliative care, psychosocial aspects of care and ethical issues. Sessions were held in English or Spanish, with simultaneous translation being provided in the main auditorium. These sessions gave participants the opportunity to hear about developments in different parts of the world. The session on perinatal and neonatal palliative care had presenters from Brazil, USA, United Kingdom, Argentina, and Uruguay, all of which are at a different phase of development in perinatal palliative care, thus giving participants an opportunity to see and understand different models of care. Psychosocial aspects of care are important, and participants presented in a mixture of Spanish and English on areas such as grief and bereavement, siblings, the compassionate mind, end of life care, the public health approach and limiting therapy at the end of life.

The theme of perinatal palliative care continued into the final plenary session for the day. The life span of infants with life-limiting and life-threatening conditions may be minutes, weeks, months or years, and, however, long or short, care must be tailored to the individual needs of the infant and their family. A case study from the care of a neonate in the United Kingdom, demonstrated the large number of professionals involved in the care of neonates, and the arising ethical issues. These ethical issues were also raised in a presentation on genetics and its implications for palliative care. Most advances in the field of genetics have occurred in areas related to diagnosis and prediction rather than treatment, yet for us working in children’s palliative care, genetic conditions play a large part in the care required.

The second day of the conference started with early morning ‘Meet the Expert’ sessions. Experts feared that they would be sitting in the venue on their own; however, many delegates turned up early to discuss bereavement, challenging communication and access to medication, and plied the experts with questions, encouraging them to share their experience and lessons learnt. The morning plenary addressed the issue of the need for a human rights perspective to children’s palliative care. Under international human rights law, countries have a legal obligation to take reasonable steps to overcome policy barriers to the provision of palliative care, yet many have not yet done so. The implications of this was discussed through case studies of children and their families. In order for children’s palliative care to develop further, education is essential. Thus, training programmes available on children’s palliative care and the principles being covered within training across the continuum of care and for a wide range of carers, such as a community care worker, and health professionals, were shared. Prior to the coffee break, the award winning ‘Little Stars’ documentary (http://www.littlestars.tv), produced by Moonshine Movies, was screened, showing the life-affirming stories of children from around the world living with life-limiting conditions and receiving palliative care support. After the screening, there was not a dry eye in the house and delegates were encouraged to use the film for the advocacy of children’s palliative care in their countries.

Concurrent sessions were held addressing a wide range of issues, including spiritual care, non-pharmacological interventions, complications of neurogenic bladder, location of care, place of death of children undergoing haematopoietic stem cell transplantation (HSCT), the development of an outcome scale for children, and the experiences of professionals and families in different settings. The morning culminated in a plenary session on the treatment of neuropathic pain and the use of adjuvant analgesia in children. It was emphasised how the management of different types of pain differs and that children may experience more than one type of pain at any one time. The multimodel treatment of neuropathic pain was discussed along with the use of integrative treatment modalities and the importance of rehabilitation, psychology, and sleep hygiene.

Concurrent sessions after lunch covered education and training along with transitions, with experiences shared from Spain, Brazil, Argentina, United Kingdom, Australia, and Ireland. Alongside these presentations were an advocacy workshop ‘*From expert practitioners to passionate advocates*’ which explored the different fora, both nationally and internationally for advocating for children’s palliative care, with particular emphasis on the WHA resolution and the recent resolutions on the control of narcotic analgesia.

For many, the highlight of the conference was the Big Debate which took place in the afternoon of the second day. The topic for debate was ‘Decision-making at the end of life’ and included emotive issues such as euthanasia and physician assisted suicide for children. Firstly, data were shared from the Netherlands which showed that most infants younger than 12 months died because life-sustaining treatment was withheld or withdrawn, usually in situations where death was imminent. The publication of the Groningen protocol in the New England Journal of Medicine in 2005 [14] generated international controversy and forced doctors to review existing approaches in palliative care in many countries, with the Dutch approach of giving lethal medication to end life. The presenter noted that legalisation of (newborn) euthanasia does not necessarily lead to widespread misuse. Secondly, on the other side of the debate, there was discussion about the concepts of compassion, personal autonomy, and utility and how these concepts can often be used in the debate around euthanasia. It was emphasised that euthanasia is not palliative care [15–16] and that the important question is not ‘What good will this child’s continuing existence do anyone?’ but ‘How best can we care for this vulnerable and suffering child?’ Through compassion and expertise, palliative care sets out to minimise the suffering of a child’s dying by maximising the pleasure in their living. As can be expected, feelings and emotions were strong and delegates had the opportunity to state their thoughts and beliefs, as well as ask questions of the speakers and question their arguments. While no vote was taken in the debate, it gave participants the opportunity to hear arguments from both sides and to be able to respond in future to questions about this sensitive area.

The final day of the conference again started with the ‘Meet the Expert’ sessions, exploring the issues of managing complex pain, fundraising and advocacy, with participants eager to engage in discussion on these issues. Prior to the morning coffee break concurrent sessions were held exploring issues of transitions and the management of pain and other symptoms. Alongside these presentations was a workshop on electronic communication that focused on utilising the media and social networking to promote children’s palliative care. This was well attended and participants engaged with the challenges and opportunities of the media and social networking.

The conference drew to a close with the final plenary session exploring the implementation of palliative care for children from the time of diagnosis or just for end-of-life care. Discussing the advantages of instituting palliative care early on, it was acknowledged the reality of many programmes having limited resources with which to care for a large number of children, thus restricting care to the end of life. Finally, the important but often neglected aspect of spirituality in children’s palliative care was addressed. The importance of providing spiritual support from the beginning of palliative care provision was stressed, recognising that often misunderstanding exists between spirituality and religion, or spiritual care and proselytising. The basic components of spiritual care were explored, drawing on current research and global thinking in this area.

In her address at the final plenary of the conference Joan Marston, CEO of the ICPCN, spoke of the need to work together and collaborate in order to continue to move children’s palliative care around the world. She urged participants to support the commitment of Buenos Aires (Figure 1) committing to share what we can and work together to achieve our global vision of palliative care for all children who need it. As the conference drew to a close delegates were reminded that for many children—there is only now and that they should continue to promote and advocate for the provision of palliative care for all children.

## Conclusion

While there has been much progress in the development of palliative care for children in the last few years, there is still a long way to go in order for the WHA resolution to be fulfilled, to ensure access to palliative care for all children around the world who need it. This second international conference convened by the ICPCN served as a unique opportunity to share with others working in the field, bringing delegates together who otherwise might not meet. While situations differ around the world, there is much that can be learnt from each other and participants took the opportunity to do that and to strive towards the further development of children’s palliative care. The conference venue was abuzz with chatter and activity during the tea and lunch breaks with delegates mixing, viewing posters, and enjoying the local cuisine [17]. The feelings engendered by the conference were captured by one delegate who wrote ‘*Congratulations to the organisation. My heart is full of enthusiasm, positive energy, and love*’.

## Conflict of Interest

The authors declare that there is no conflict of interest.

## Figures and Tables

**Table 1.** Key issues addressed in the pre-conference workshops.

Pain in children	Preventing and treating pain in infants and children with life-limiting diseases. Why we need to assess pain in children and how it is best done, addressing some of the common myths about paediatric pain and its measurement, along with describing the knowledge and skills needed to accurately assess a child’s pain. How to treat pain in children, reviewing assumptions about opioid use and evaluating the WHO principles of pain management [10–11]. Complex pain in children, including psychological, social and spiritual pain and physical pain. Management of neuropathic pain and the use of adjuvant analgesia.
Adolescent palliative care	Defining adolescence and exploring some of the key issues for the provision of palliative care for this age group, such as developmental stages, the uniqueness of the individual, their perceived loss of control, concerns about body image, the need to explore boundaries, and the importance of their peer group. Palliative care for adolescents with cancer including some of the key issues faced by them, such as body image, sexuality, etc. The needs of adolescents with non-malignant diseases and the impact of having been unwell for many years on the understanding of their illness, death, and dying. Issues around transition from childhood to adulthood and from children’s to adult services, exploring both the personal transitions faced and those connected to service provision. The importance of psychological and spiritual care
Principles of CPC	Exploring the basic principles of palliative care for children. Management of pain in children and control of other symptoms. Communication in CPC. Palliative care emergencies.
Invisible aspects of CPC	The role of an odontologist in the palliative care team and general aspects of oral care. The challenges posed by disease progression. What we do and what we do not communicate. Issues around adherence to treatment and attention to detail in prescribing in palliative care for children.
Ethics/Decision-making	Parental authority. Perinatal palliative care. ‘Futile’ treatment. When and what to tell the child or their parents.
Developing programmes	Sharing experiences from different settings, e.g., China, Belarus, and India. Exploring why the different programmes had developed, i.e., the need, the way that the programmes were developed, and the problems that were encountered and how they were overcome.

**Table 2.** Countries represented at the conference.

1. Argentina 2. Australia 3. Austria 4. Bangladesh 5. Belgium 6. Belarus 7. Bolivia 8. Brazil	9. Canada 10. Chile 11. Columbia 12. Costa Rica 13. Dominican Republic 14. Ecuador 15. Germany 16. Guatemala	17. India 18. Ireland 19. Italy 20. Japan 21. Kenya 22. Malawi 23. Mexico 24. The Netherlands	25. New Zealand 26. Paraguay 27. Peru 28. Romania 29. Russia 30. South Africa 31. Spain 32. Swaziland	33. Sweden 34. Switzerland 35. Taiwan 36. United Kingdom 37. Uganda 38. Uruguay 39. United States of America 40. Zimbabwe.

**Figure 1.** Commitment of Buenos Aires.

We believe that all children with life-threatening and life-limiting conditions have the right to receive quality palliative care provided by trained professionals and support workers, wherever they live in the world. We are concerned about the large and unmet needs of children requiring palliative care, especially in low- and middle-income countries where the need is greatest. We call on all governments to implement the World Health Assembly Resolution 67. On 19th May 2014 on palliative care to ensure equitable access to palliative care, including pain relief, for neonates, children and young people and their families. As palliative care practitioners and advocates, we recognise that disparities exist within and between countries and services, but collectively, we are a rich resource of knowledge and skills. We therefore commit to share all that we can and to collaborate with the World Health Organisation (WHO), UNICEF, governments, and other relevant groups to achieve our global vision of palliative care for all children who need it.
